# Lower Incisor—Pg: A New Cephalometric Parameter to Evaluate the Anterior Limit of Dentition

**DOI:** 10.3390/dj11110264

**Published:** 2023-11-10

**Authors:** Marzio Galdi, Federica Di Spirito, Alessandra Amato, Davide Cannatà, Roberto Rongo, Stefano Martina

**Affiliations:** 1 Department of Medicine, Surgery and Dentistry “Scuola Medica Salernitana”, University of Salerno, Via Allende, 84081 Baronissi, Italyfdispirito@unisa.it (F.D.S.);; 2 Department of Neurosciences, Reproductive Sciences and Oral Sciences, University of Naples Federico II, Via Pansini 5, 80131 Naples, Italy

**Keywords:** lower incisor position, facial aesthetic, cephalometric, epidemiology, orthodontics, diagnosis, treatment, anterior limit of dentition

## Abstract

*Background*: This present retrospective study aimed to introduce Lower Incisor—Pg and to assess how this new parameter varies with the skeletal sagittal and vertical relationships. *Methods*: A total of 1256 cephalometric analyses were performed using lateral cephalometric radiographs of a sample of subjects divided according to *SN^Go-Gn*, *ANPg^*, and *IMPA* measurements. The differences in *Lower Incisor—Pg* between the groups were assessed through ANOVA tests and posthoc analyses, while Pearson’s correlation analysis was used to assess the correlations between the measurements. *Results*: The mesofacial growth pattern (61.0%) was more common than dolichofacial (30.0%) and brachyfacial (8.6%) ones in the sampled population. Regarding skeletal sagittal relationships, Class I was more frequent (70.9%) than Class II (19.3%) and Class III (9.8%). The mean value of the Lower Incisor—Pg was 3.2 ± 4.0 mm. Linear regression showed that the β coefficient was 0.45 and 0.36 for ANPg^ and SnGoGn^, respectively. *Conclusions*: Lower Incisor—Pg is a linear cephalometric measurement to evaluate the lower incisor sagittal position. For each degree of increase in ANPg^ and SNGoGn^, the Lower Incisor—Pg increased 0.45 mm and 0.36 mm, respectively.

## 1. Introduction

Facial aesthetics and functional harmony have been recognized to be of prime importance at the time of orthodontic diagnosis and treatment planning [1]. Although facial beauty is a subjective concept [2], objective measurements have been correlated with the perceived facial appearance and, thus, should be considered complementary to the clinical experience when planning orthodontic therapies [3]. The lower incisors probably represent the most crucial dental elements in terms of facial aesthetics and function. The sagittal position of the lower incisors is closely related to both facial appearance and functional harmony [1]. A correlation exists between the sagittal lower incisor movements and lip position and thickness since lower incisor retraction results in lower lip retraction and decreased lip thickness.

Furthermore, the sagittal position of the lower incisors is influenced by both vertical and sagittal skeletal relationships [4]. Regarding the vertical skeletal variation, the lower incisors are usually more retroclined in dolichofacial phenotypes and more proclined in brachyfacial ones [5]. Regarding the sagittal skeletal relationship, the incisor position tends to compensate for skeletal discrepancies. Regarding gingiva and periodontium, an excessive lower incisal inclination can cause a gingival recession, bone dehiscence, and fenestration in the presence of a thick gingival phenotype [6,7,8].

Lastly, the planning of the lower incisor position is affected by the need to create space within the lower dental arch to solve anterior dental crowding [9]. In fact, the movement of the lower incisors represents the most easily implemented non-invasive strategy to gain space within the dental arch since the other strategies (distalization and expansion) are limited by the mandibular anatomy [10]. Because the maxilla does not have similar anatomical limitations, the position of the upper incisor can be subsequently adapted to that established for the lower incisor [11].

Since the lower incisor and its position in the lower arch assumed a key role in orthodontic diagnosis and treatment planning [12], different cephalometric methods have been proposed to establish the correct lower incisor position [5].

Margolis introduced the “Incisor-Mandibular Plane Angle” (IMPA), which is described as the angle between the lower incisor axis and the mandibular plane [13].

Later, Tweed pointed out an important limitation of the angle described by Margolis, whose value was heavily influenced by mandibular morphology. He proposed a diagnostic triangle including the FMIA, FMA, and IMPA to also consider vertical skeletal discrepancies in the prognosis of the lower incisor position [14,15].

Ricketts related the position of the lower incisor to upper cranial structures, therefore introducing an angular cephalometric parameter, which is the angle between the axis of the lower incisor and the Frankfurt plane (B1 to FH). The Frankfurt plane is parallel to the horizontal plane; thus, FH inclination is constant and B1 to FH only depends on B1 inclination [16].

The most widely used parameter in assessing the position of the anterior limit of the dentition remains the IMPA, which, however, has limitations in the evaluation of subjects with increased verticality or major sagittal discrepancies [17]. Other linear parameters such as A-Pog by Ricketts and NB by Steiner [1] are affected by the position of the maxilla and upper cranial structures. Considering this, the aims of this study were to introduce a new linear measurement for the evaluation of the lower incisor position, named Lower Incisor—Pg, which could better interpret the relation between the anterior limit of the dentition and the vertical and sagittal pattern of the subjects, and to assess how the new parameter measurement varies with the skeletal sagittal and vertical relationships.

Therefore, a preventive analysis of the cephalometric values of the skeletal sagittal and vertical relationships and incisor position in a population of subjects, divided according to age and sex, was performed.

## 2. Materials and Methods

This was a retrospective study that analyzed the original lateral cephalometric radiographs of 1836 subjects, who were recruited at the Section of Orthodontics at the University of Naples Federico II in Naples, Italy. All the radiographs were transferred anonymously, with only the patients’ ages and genders being recorded.

The inclusion criteria were as follows:

- Age of subjects: radiographs of subjects older than 8 in order to ensure that the central incisors were erupted at the time they were taken (mean age of central incisor eruption 6 ± 12 months) [18];
- Quality of radiographs: high-quality lateral cephalometric radiographs.

Instead, exclusion criteria were as follows:

- Age of subjects: radiographs of subjects younger than 8 years old;
- Previous treatment: radiographs of subjects with implants or prosthetic framework and those of subjects who underwent orthodontic treatment;
- Quality of radiographs: poor-quality teleradiographs (splitting of the image due to patient movement or malposition; teleradiographs in which the central incisors were not clearly identified).

Furthermore, when two lateral cephalometric radiographs of the same patient were available, notably one in habitual occlusion and the other in centric occlusion, the choice always fell on the second one.

The sample was divided into two groups according to age:

- Growing group: subjects aged between 8 and 18 years old;
- End-growth group: subjects older than 18 years old.

One trained examiner (M.G.) independently performed a cephalometric analysis of the lateral cephalometric radiographs using Delta-Dent software 2.0 (Delta-Dent CE—Outside Format, Pandino, CR, Italy) with a standardized calibration. Where there was a double projection of two points, the midpoint was used.

**Figure 1:**
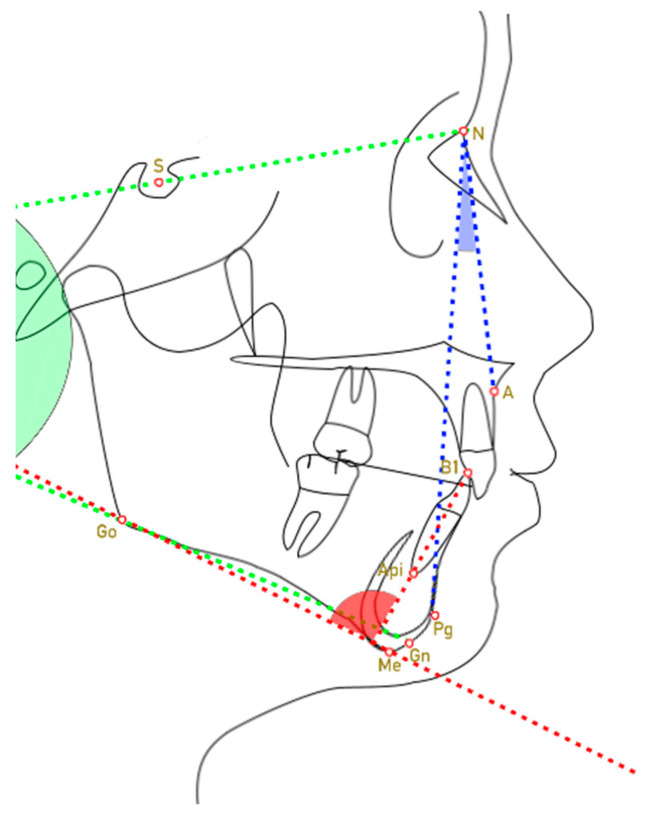
Parameters included in the cephalometric analysis. The angle marked by the blue lines is the ANPg^. The angle marked by the green lines is SN^Go-Gn. The angle marked by the red lines is IMPA.

The points identified in the cephalometric analysis are described in Table 1 and shown in Figure 1.

- The vertical and sagittal skeletal relationships of subjects in each group were evaluated by analyzing, respectively;
- SN^Go-Gn: angle formed by the N-S plane with the Go-Gn plane;
- ANPg^: angle formed by Point A, Nasion, and Pogonion.

The lower incisor position was determined through IMPA measurements, which is the angle formed by the axis of the lower incisor and the mandibular plane (Go-Me).

According to the cephalometric measurements obtained, the sample was divided as follows:

- Based on the skeletal vertical relationship, the sample was divided into mesofacial, brachyfacial, and dolichofacial groups, defined according to the SN^Go-Gn. Notably, subjects were considered mesofacial, brachyfacial, and dolichofacial when the SN^Go-Gn was between 27° and 37°, lower than 27°, and greater than 37°, respectively [19].
- Based on the skeletal sagittal relationship, the sample was divided into Class I, Class II, and Class III groups, defined according to the ANPg^. Specifically, subjects fell into Class I group when ANPg^ was between −1° and 5°, Class II group when ANPg^ was greater than 5°, and Class III group when ANPg^ was less than −1° [19].
- Based on incisor position, the sample was divided into normoclined, proclined, and retroclined incisor groups, defined according to the IMPA. Particularly, the incisors were considered normoclined when IMPA was 90° ± 5°, proclined when IMPA was greater than 95°, and retroclined when IMPA was less than 85°.

**Figure 2:**
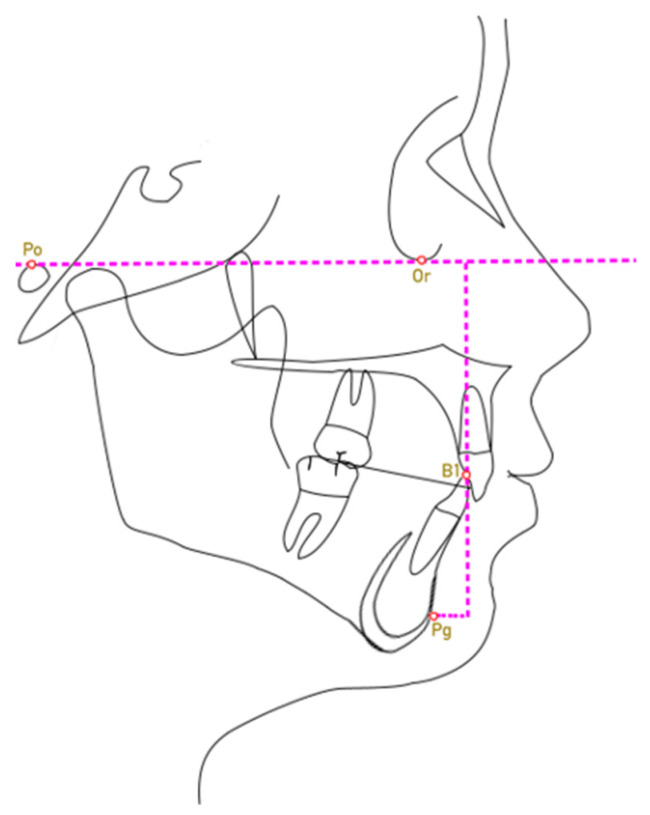
Lower Incisor—Pg parameter.

Then, the linear distance between the line perpendicular to the Frankfurt plane passing through the incisal margin of the lower incisor and the Pogonion (Lower Incisor—Pg; Figure 2) was calculated for the subjects in each group.

The mean and standard deviation of quantitative data were calculated. A Shapiro–Wilk test was performed to assess if quantitative variables were normally distributed in each group.

A z-test was performed to compare the means of values between the growing and end-growth groups and between males and females.

Analysis of variance (ANOVA) tests and posthoc analysis were performed to assess the differences in the Lower Incisor—Pg measurements between the brachyfacial, mesofacial, and dolichofacial groups, between the Class I, Class II, and Class III skeletal groups, and similarly between the normoclined, proclined, and retroclined incisor groups. Pearson’s correlation analysis was used to measure the correlation of the Lower Incisor—Pg measurements and ANPg^, SN^GoGn, and IMPA. Linear regression analysis was carried out to assess how the Lower Incisor—Pg changed according to ANPg^, SN^GoGn, and IMPA. A standard statistical program (SPSS, version 28.0; IBM SPSS, Armonk, NY, USA) was used. The significance level was set at *p* < 0.05.

## 3. Results

A total of 1256 subjects (684 females and 572 males), aged between 8 and 57, met the eligibility criteria and, therefore, were included in this present study.

The cephalometric measurements are reported in Table 2.

The skeletal sagittal measurements, skeletal vertical measurements, and the IMPA were normally distributed among the male and female groups, and the SN^Go-Gn significantly differed between the two groups (*p* < 0.001) (Table 2).

When considering the growing group and the end-growth group, involving 1009 and 247 subjects, respectively, the SN^Go-Gn, ANPg^, and IMPA measurements were normally distributed. Statistically significant differences between the two groups were found in ANPg^ (*p* < 0.001) and IMPA (*p* < 0.011).

Cephalometric measurements of growing and end-growth subjects are reported in Table 3.

The mean value of the Lower Incisor—Pg was 3.2 ± 4.0 mm. The parameter was normally distributed in the sample and did not statistically differ between the male and female groups (*p* = 0.213). There was a statistically significant difference between age groups (*p* < 0.001).

The Lower Incisor—Pg showed statistically significant differences between groups of subjects divided according to the skeletal vertical and sagittal relationship and IMPA measurements, as Table 4 shows.

**Figure 3:**
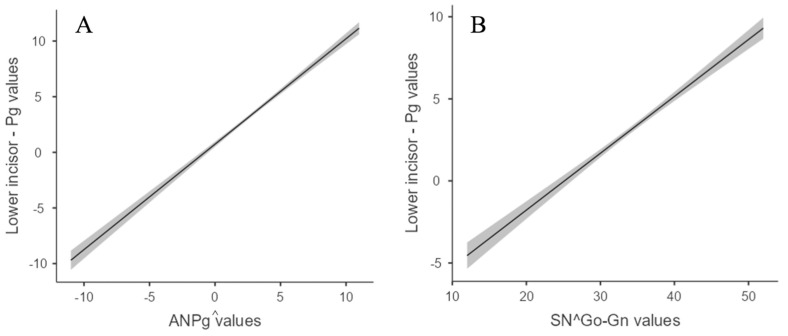
Scatter plot displaying linear regression between Lower Incisor—Pg values and sagittal (**A**) and vertical (**B**) skeletal relationship values.

The relation between the Lower Incisor—Pg and the ANPg^ and SNGoGn^ angles was strong (Pearson’s r, r = 0.792, *p* < 0.001). Linear regression between these variables showed that the β coefficient was 0.45 and 0.36 for ANPg^ and SnGoGn^, respectively, as shown in Figure 3. A linear correlation was absent between the Lower Incisor—Pg and age (Pearson’s r, r = 0.10, *p* < 0.001) in the group of all subjects, as well as in the group of growing subjects only (Pearson’s r, r = 0.086, *p* = 0.006).

## 4. Discussion

This study found that the mesofacial growth pattern (61.0% of subjects) was more common than the dolichofacial (30.0%) and brachyfacial (8.6%) ones in the sampled population of subjects from Southern Italy. Regarding skeletal sagittal relationships, Class I was more frequent (70.9%) than Class II (19.3%) and Class III (9.8%).

The comparison of these findings to data reported in the scientific literature is possible only when considering studies that took a population from the same geographic area as their sample since craniofacial morphology is affected by race [20].

Studies investigating skeletal vertical and sagittal cephalometric parameters of populations from different regions of the world showed deeply heterogeneous findings. However, results from a retrospective study by D’Antò et al. [19] are consistent with those of this present study, as the samples are comparable in provenance.

The prevalence of skeletal Class I and Class II found in this present study matches the prevalence of dental Class I and Class II observed in previous epidemiological studies [21]. The frequency of skeletal Class III observed in our sample does not reflect the presence of dental Class III reported in epidemiological studies [22]. The main reason for this could be the dental compensation that may mask a mild skeletal Class III and could also influence the position of cephalometric points A and B [21].

When comparing the male and female groups, statistically significant differences were found in the SN^Go-Gn. Consistently, studies on the craniofacial growth changes showed a net rotation of the jaws in a forward direction in male subjects, slightly decreasing the mandibular plane angle. Instead, females seemed to have a tendency toward a backward rotation, with an increase in the mandibular plane angle [23].

When comparing the growing and end-growth groups, the mean value of the ANPg^ was significantly lower in the end-growth groups. This result can be easily understood, considering that the beginning and ending of sagittal growth of the mandible are more delayed than the beginning and ending of sagittal maxillary growth [24]. Accordingly, the prevalence of skeletal Class III in growing subjects was 7.8% (n = 79 out of 1009), while in the end-growth groups, it was 17.8% (n = 44 out of 247).

Furthermore, the mean value of the IMPA statistically differed between growing and end-growth subjects, slightly decreasing in adults. The normal rotation of the mandible during growth tends to alter the eruption path of the incisors, tending to upright the incisors and lingually positioning them [25].

Lower Incisor—Pg can be described as the distance between the line perpendicular to the Frankfurt plane passing through the incisal margin of the lower incisor and the Pogonion. Since this distance only depends on the incisal margin position and the Pogonion, the angle of the mandibular plane (Go-Me) does not affect this parameter, exceeding the main limitation of the IMPA measurement. Indeed, the IMPA value is deeply affected by the angle of the mandibular plane.

**Figure 4:**
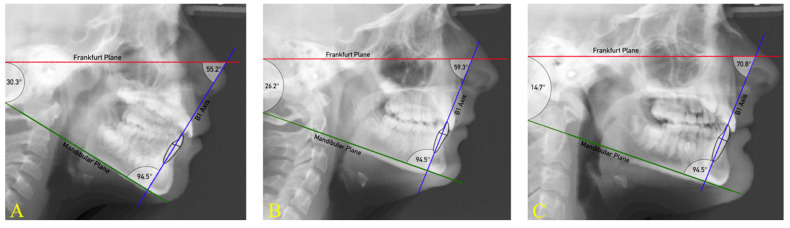
According to Tweed’s definition, a triangle formed by the intersection of the Frankfurt plane (red line), the mandibular plane Go-Me (green line), and the lower incisor (B1) axis (blue line) has been identified. The angles subtended between these planes are as follows: IMPA (mandibular plane to B1 axis), mandibular plane angle (Go-Me to FH), and B1 to FH. The three different subjects have the same IMPA, but different mandibular plane angle: subject **A** is hyperdivergent, subject **B** is normodivergent, and subject **C** is hypodivergent. Even though the IMPA is the same, the angle B1 to FH is increased as the mandibular plane angle is decreased.

Figure 4 shows three lateral cephalometric radiographs from this present study sample. When considering B1 to FH, the incisor of the subject in teleradiograph A is more proclined than for the subjects in radiographs B and C. However, the IMPA value of the subjects is the same because the angle of the mandibular plane of the three subjects was different. Thus, evaluating the real position of the lower incisor through the IMPA is only possible by relating this measurement to the angle formed by the mandibular plane Go-Me and FH (angle of mandibular plane) [26].

**Figure 5:**
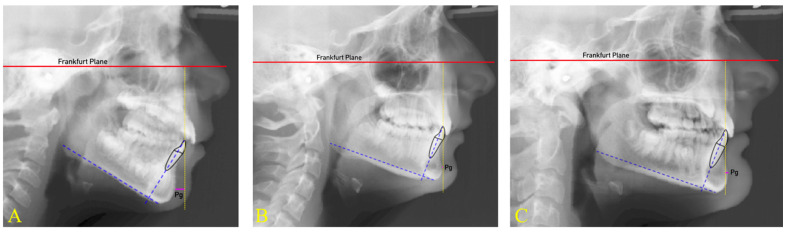
The change in the angle B1 to FH shown in Figure 4 corresponds to the change in the Lower Incisor—Pg (purple line); the distance between Pg and the line perpendicular to FH (red line) passed through the incisal margin of the lower incisor (yellow line). Subject **A** has an increased value of Lower Incisor—Pg, subject **B** has a normal Lower Incisor—Pg, subject **C** has a decreased Lower Incisor—Pg.

Figure 5 shows the same radiographs as Figure 4. In contrast to the IMPA value that is constant, the variation in Lower Incisor—Pg measurements between the subjects reflects changes in the inclination of B1 to FH, confirming that the angle of the mandibular plane does not affect this parameter.

The mean value of the Lower Incisor—Pg in this present studied sample was 3.2 ± 4.0 mm. As the value of IMPA, the Lower Incisor—Pg did not statistically differ between the male and female groups, whereas there were significant differences between the growing and end-growth groups.

Even if the Lower Incisor—Pg was not affected by the inclination of the mandibular plane, statistically significant differences were found between groups of subjects divided according to skeletal vertical and sagittal relationships. This is because the Lower Incisor—Pg measurement is affected by the position of the Pogonion, which depends on the vertical and sagittal position of the mandible.

However, changes in the Lower Incisor—Pg, depending on the position of the mandible, are predictable. Indeed, linear regression between the Lower Incisor—Pg, ANPg^, and SNGoGn^ showed:

The Lower Incisor—Pg was increased by 0.45 mm for each one-degree increase in the ANPg^.

The Lower Incisor—Pg was increased by 0.36 mm for each one-degree increase in the SNGoGn^.

The same can be applied to other cephalometric parameters in the literature used to assess the position of the lower incisor. Ricketts’ A-Pg line [1], for example, is strongly influenced by the position of the upper limit of the dentition, whereas Steiner’s N-B [1] is related to the position of the N point and the position of the B point, which could be affected by the inclination of the lower incisor. In view of these considerations, The Lower Incisor—Pg might be a more reliable parameter to be used by clinicians in cephalometric analysis as it is not affected by Nasion and maxillary positions and can read mandibular plane changes better than IMPA. Despite advances in digital and three-dimensional diagnostics, cephalometric analysis remains an essential tool in orthodontic diagnosis [27], thanks also to the recent possibility of identifying landmarks with artificial intelligence [28]. In particular, it is of crucial importance to determine the position of the anterior limit of the dentition, which is the key factor in treatment planning [29]. Hence, Lower Incisor—Pg could provide additional information in planning orthodontic movements and visualizing treatment objectives.

This present study has a few limitations: it is a retrospective study that analyzed a lower incisor position only based on cephalometric measurements. In clinical settings, when evaluating the desired position of the anterior limit of dentition, practitioners should always consider clinical features, such as the gingival phenotype and the risk of gingival recessions, which can limit the possibility of moving the lower incisor. However, given that the sample size has no equal in previous studies, in addition to its heterogeneity, the results obtained can be a valuable tool for orthodontists in diagnosis and treatment planning.

## 5. Conclusions

The analysis performed showed the following:

- In the studied sample, the Lower Incisor—Pg presented a mean value of 3.2 ± 4.0 mm, and it was not influenced by the angle of the mandibular plane.
- For each degree of increase in ANPg^ and SNGoGn^, the Lower Incisor—Pg increased, respectively, by 0.45 mm and 0.36 mm.

## Figures and Tables

**Table 1:** Cephalometric points.

Cephalometric Point	Description
Nasion (Na)	the most anterior point of the nasofrontal suture
Menton (Me)	the lower midpoint located on the inferior curve of the symphysis
Sella turcica (S)	the midpoint of the sella turcica
Orbitalis (Or)	the lowest point of the orbital cavity
Porion (Po)	the posterosuperior margin of the external auditory meatus
Pogonion (Pg)	the most anterior point of the mandibular symphysis
Subspinal point (A)	the most posterior point of the anterior concavity of the maxilla, between the anterior nasal spine and the alveolar processes
Gnathion (Gn)	the midpoint between pogonion and menton
Incisal margin of the lower incisor	the point on the incisal margin of the lower incisor
Root apex of the lower incisor	the point on the apex of the lower incisor

**Table 2:** Cephalometric measurements of skeletal vertical and sagittal relationships and incisor positions in the sample divided according to sex.

Cephalometric Measurements	N(%)	Mean ± SD Value of Subjects (mm)	Mean ± SD Value of Female Subjects (mm)	Mean ± SD Value of Male Subjects (mm)	*p*-Value *
SN^Go-Gn			35.2 ± 5.6	33.3 ± 5.5	<0.001
Mesofacial	766 (61.0%)	32.7 ± 3.0			
Dolichofacial	381 (30.3%)	40.2 ± 4.1			
Brachyfacial	109 (8.7%)	25.1 ± 4.0			
ANPg^			2.7 ± 2.6	2.5 ± 2.9	0.193
Skeletal Class I	890 (70.9%)	2.3 ± 1.8			
Skeletal Class II	243 (19.3%)	5.8 ± 1.9			
Skeletal Class III	123 (9.8%)	−1.9 ± 2.4			
IMPA			95.5 ± 7.4	96.1 ± 7.9	0.147
Normoclined	484 (38.5%)	91.6° ± 4.3			
Proclined	680 (54.1%)	100.6° ± 5.3			
Retroclined	92 (7.3%)	82.4° ± 6.1			

* Independent z-test, significant at *p* < 0.05.

**Table 3:** Cephalometric measurements of skeletal vertical and sagittal relationships and incisor positions in the sample divided according to age.

Cephalometric Measurements	Mean ± SD Value of Growing Group (mm)	Mean ± SD Value of End-Growth Group (mm)	*p*-Value *
SN^Go-Gn	34.2 ± 5.3	33.9 ± 6.9	0.938
ANPg^	2.8 ± 2.7	1.7 ± 2.9	<0.001
IMPA	96.1 ± 7.5	94.6 ± 8.0	0.011

* Independent z-test, significant at *p* < 0.05.

**Table 4:** Lower Incisor—Pg measurements in the sample divided according to skeletal vertical and sagittal relationships and IMPA measurements.

Group of Subjects	Lower Incisor—Pg Mean ± SD (mm)	*p*-Value *
Mesofacial	2.7 ± 3.6	<0.001
Dolichofacial	5.2 ± 3.9	
Brachyfacial	−0.5 ± 3.6	
Class I	2.9 ± 3.5	<0.001
Class II	6.3 ± 3.7	
Class III	−0.9 ± 3.9	
Normoclined incisor	2.4 ± 3.9	<0.001
Proclined incisor	4.2 ± 3.8	
Retroclined incisor	−0.3 ± 3.7	

* ANOVA test, significant at *p* < 0.05.

## Data Availability

Data are available upon request to the corresponding author.
